# Effect of a home-based exercise program on functional mobility and quality of life in elderly people: protocol of a single-blind, randomized controlled trial

**DOI:** 10.1186/s13063-018-3061-1

**Published:** 2018-12-12

**Authors:** Glauber Sá Brandão, Luís Vicente Franco Oliveira, Glaudson Sá Brandão, Anderson Soares Silva, Antônia Adonis Callou Sampaio, Jessica Julioti Urbano, Alyne Soares, Newton Santos Faria, Luisa Teixeira Pasqualotto, Ezequiel Fernandes Oliveira, Rodrigo Franco Oliveira, Deise A. A. Pires-Oliveira, Aquiles Assunção Camelier

**Affiliations:** 1 Bahia School of Medicine and Public Health, Salvador, BA Brazil; 2 Department of Education (DEDC-VII), Bahia State University – UNEB, Senhor do Bonfim, BA Brazil; 3 Medical School, Centro Universitário de Anapolis – UniEVANGÉLICA, Av. Universitária Km 3,5 – Cidade Universitária, Anápolis, GO Brazil; 4 Diagnostic and Specialty Clinic – IMAIS, Senhor do Bonfim, BA Brazil; 5 Rehabilitation Sciences Master’s and Doctoral degree, University of Nove de Julho – UNINOVE, São Paulo, SP Brazil; 6 Physiotherapy School, Nove de Julho University – UNINOVE, São Paulo, SP Brazil; 7 Physiotherapy School, University of the State of Minas Gerais – UEMG, Divinópolis, MG Brazil

**Keywords:** Aged, Motor activity, Quality of life, Community

## Abstract

**Background:**

Elderly people have high rates of functional decline, which compromises independence, self-confidence, and quality of life (QoL). Physical exercise leads to significant improvements in strength, balance, functional mobility, and QoL, but there is still reduced access to this therapeutic strategy due to difficulties in locomotion to training centers or lack of adaptation to the exercise environment.

**Methods/design:**

The purpose of this clinical trial will be to verify the effect of a progressive and semi-supervised, home-based exercise program on the functional mobility, and in the QoL of sedentary elderly people. This is a protocol of a consecutive, single-center, single-blind, and randomized controlled trial. The design, conduct, and report follows the SPIRIT (Standard Protocol Items: Recommendations for Interventional Trials) guidelines. Sedentary elderly people will be enrolled, and randomly allocated into two groups. The intervention group will perform exercises in their own home and the control group will not perform exercises. The evaluations will occur at study enrollment and after 3 months of intervention, and will be performed using the functional mobility Timed Up & Go (TUG) test and sociodemographic and QoL questionnaires. In the statistical analysis, comparisons of mean and correlation analyses will be performed. The primary expected outcome is the improvement in functional mobility verified through the TUG test and the secondary outcome is the improvement in QoL verified by the WHOQOL-OLD.

**Discussion:**

The lack of scientific evidence demonstrating the benefits of semi-supervised home exercise on functional mobility and QoL in elderly people represents an obstacle to the development of guidelines for clinical practice and for policy-makers. The World Health Organization highlighted the importance of musculoskeletal health programs for elderly people, and the exercise program described in this protocol was designed to be viable, easy to implement, and inexpensive, and could be performed at the home of elderly subjects after receiving only guidelines and follow-up via periodic visits. Based on these facts, we hope that this study will demonstrate that a well-structured, home-based exercise program can be effective in improving functional mobility and QoL of sedentary elderly people, even without constant supervision during exercise.

**Trial registration:**

Registro Brasileiro de Ensaios Clínicos (ReBEC), Identifier: RBR-3cqzfy. Registered on 2 December 2016.

**Electronic supplementary material:**

The online version of this article (10.1186/s13063-018-3061-1) contains supplementary material, which is available to authorized users.

## Background

Increased life expectancy is associated with biopsychosocial changes that occur naturally with advancing age [1]; among these changes, the decline in functional performance, and fear of falls are some of the factors with the most influence on quality of life (QoL) in elderly people [2, 3]. The growing process of population aging has emerged as a clinical priority in the fields of public health and social assistance policies [4], considering that old age and disability are among the main determinants of the use of public health services [5, 6].

Elderly people in the community have high rates of functional decline and disability, progressively compromising their independence, self-confidence, and QoL. These impairments are intensified by physical inactivity, which is linked to important negative outcomes in the general health of elderly people [7, 8]. Some studies have demonstrated that the implementation of specific physical exercise programs produces significant improvements in muscle strength, balance, and functional mobility of elderly people, even in subjects of more advanced age, and that regular practice of these exercises produces positive effects on the QoL in this population [7, 9, 10].

However, despite scientific evidence showing the benefits of physical exercise in this population, there is still low adherence to this strategy, possibly related to external factors such as difficulties in locomotion or non-adaptation to the environment where the exercises are performed, since the majority of studies use supervised exercise programs performed at physical training centers [11]. A recent study [12] showed that elderly people with a history of falls prefer to participate in exercise programs that can be performed at home or do not require transportation.

Considering that semi-supervised, home-based exercise is a safe, inexpensive, and easy-to-implement therapeutic resource, this study aims to test the hypothesis that the regular practice of a progressive physical exercise program performed at home improves functional mobility and QoL in a population of sedentary elderly individuals. Our hypothesis is that regular practice of semi-supervised and progressive home exercise improves functional mobility and QoL of sedentary community-dwelling elderly subjects. The primary objective of the study is to verify the effect of a progressive and semi-supervised, home-based exercise program on the functional mobility of a population of sedentary elderly persons in the community. The secondary objective is to analyze the effect of a progressive and semi-supervised, home-based exercise program on the QoL of a sedentary elderly population in the community.

## Methods/design

### Study design and setting

**Fig. 1:**
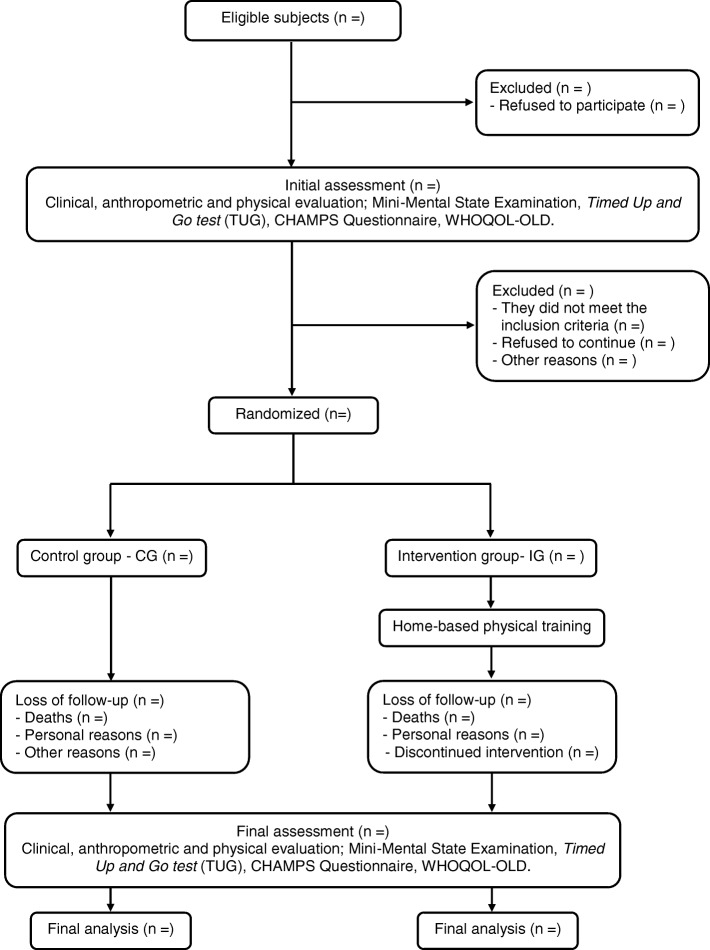
Flow chart of the study

**Fig. 2:**
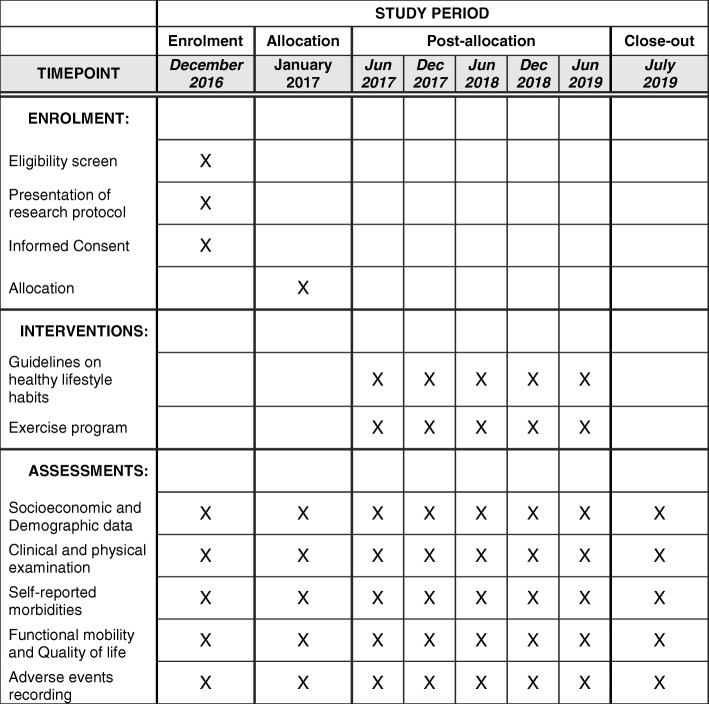
Standard Protocol Items: Recommendations for Interventional Trials (SPIRIT) diagram

The design and conduct of this study follows the guidelines of The Standard Protocol Items “Recommendations for Intervention Trials (SPIRIT) guidelines” [13], according to the flow chart of the study showed in Fig. 1. This is a blind, randomized controlled clinical trial, using a superiority test and a 1:1 allocation ratio. The sample will be of convenience, composed of elderly subjects in a community in the city of Senhor do Bonfim, BA, northeastern region of Brazil. The protocol is reported according to the Standard Protocol Items: Recommendations for Interventional Trials (Fig. 2 SPIRIT diagram and Additional file 1: SPIRIT Checklist).

### Ethical and legal aspects

This study protocol was approved by the Research Ethics Committee of the Bahia School of Medicine and Public Health (Brazil), process no. 39072514.6.0000.5544, and was registered on the website “ensaiosclinicos.gov.br with Identifier: RBR-3cqzfy.” All participants must agree and sign the informed consent form to participate in the study. Removal is permitted at any time without any loss. After the explanatory meeting with all subjects involved in the study, the informed consent will be obtained individually, in a reserved place, by the main investigator and supervising physician.

### Participants and recruitment procedure

The research will be conducted from December 2016 to July 2019, involving elderly people of both sexes, aged over 60 years old, living in the municipality of Senhor do Bonfim, BA, in the northeastern region of Brazil. Recruitment will be consecutive, across the community, from December 2016 to December 2018. Initially, it will take place through the dissemination of the research study in local newspapers, radios, religious centers, meeting groups for the elderly, senior residency, neighborhood associations, and the Third Age Project developed by the Municipal Government. In this announcement will be provided a telephone so interested people will be able to contact the team of researchers.

### Eligibility criteria

The inclusion criteria for the study will be age ≥ 60 years old and not having performed regular exercise for at least 3 months prior to the start of the study. Participants with cognitive deficits will be excluded according to the Portuguese version of the Mental State Mini Exam (MSME) (an illiteracy group ≤ 20, a primary school group ≤ 25, a junior high school group ≤ 26.5, a high school group ≤ 28, and a well-educated group ≤ 29). According to the authors, its application is very good for hospital patients, outpatients, and for the population study, including the elderly [14]. Participants will also be excluded if they have any clinical and/or orthopedic condition that contraindicates the performance of regular physical exercise, identified through a clinical and physiotherapeutic evaluation.

### Randomization and allocation concealment

All subjects involved in the study, before randomization, will participate in groups of 12 elderly in a 40-min talk with explanations on the evaluation and intervention procedures and will receive educational leaflets containing guidelines on healthy habits related to food, hydration, and sleep hygiene. The subjects in the IG will be informed that they will follow the guidelines of healthy habits of life and a program of home-based physical exercise. For this program, they will participate in theoretical-practical training concerning adequate accomplishment of the exercises and will receive a booklet, developed by the researchers, that contains illustrative and written guidelines on the method of performing the exercises, and a diary to register the frequency of exercise on a weekly basis.

After the explanatory meeting with all subjects, the main investigator will enroll the subjects of the study and request the randomization. A researcher from the same department, not involved in the trial, will prepare the group allocation after explanatory meeting and baseline assessment. Randomization will be computer based and carried out at a 1:1 ratio according to a random sequence generated by the Research Randomizer (https://www.randomizer.org/), generating two groups, control group (CG) and intervention group (IG: semi-supervised, home-based exercise program). Consecutively numbered, sealed, opaque envelopes containing group allocation will be prepared by the same researcher not involved in the trial. The concealed envelopes will be securely stored and will be opened in sequence to reveal group allocation (CG or IG).

The researchers, after making sure that the participants will be able to properly perform all the proposed exercises, will guide the family members to help the participants and stimulate their practice. Any change in the physical or mental condition of the participants should be communicated by telephone to the research team. Participants in the CG will be informed that they should only continue their daily living activities and follow the guidelines of healthy living habits.

### Clinical evaluation

A previously trained physician and physiotherapist will perform all evaluations. Participants will receive standard verbal instructions on the procedures and will be evaluated individually in an appropriate room. Evaluations will be conducted before and after the intervention period. A clinical evaluation, physical examination, and evaluation of functional mobility, and physical activity levels, and QoL will be performed and socioeconomic, demographic, anthropometric, and self-referenced morbidity data will be collected according the schedule for data collection and outcome measurement (Table 1).

**Table 1:**
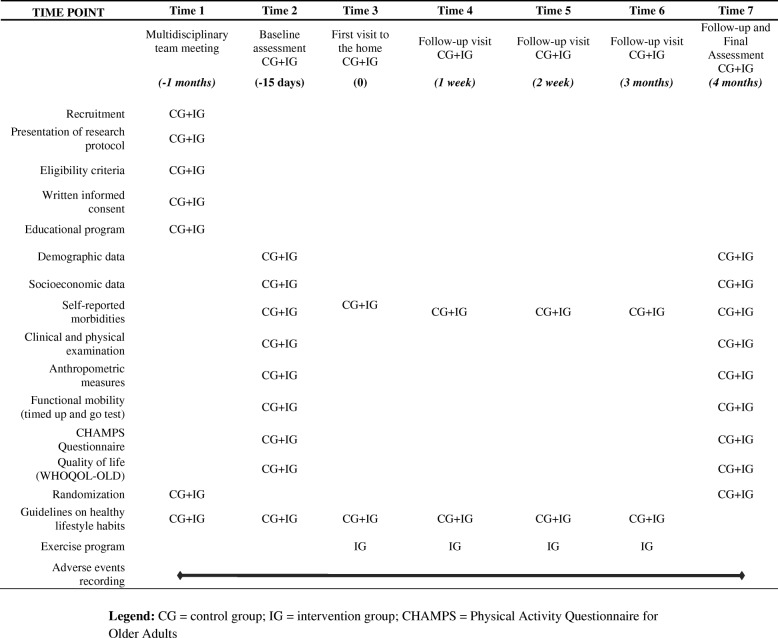
The schedule for data collection and outcome measurement

*CG* control group, *IG* intervention group, *CHAMPS* Physical Activity Questionnaire for Older Adults

Weight will be measured while wearing light clothing and without shoes, after emptying of the bladder, using a digital scale to the nearest 100 g. Height will be measured without shoes, with a stadiometer to the nearest 0.5 cm (model 200/5; Welmy Industria e Comercio Ltda, Sao Paulo, Brazil). Body Mass Index (BMI) will be calculated by dividing weight (kg) by the square of height (m) [15].

Functional mobility will be evaluated by the Timed Up & Go (TUG) test. This test measures, in seconds, the time taken by an subject to stand up from a standard arm chair (seat of the chair with height of 46 cm and arms with 65 cm of height), walk a distance of 3 m, at a comfortable and safe pace, turn, walk back to the chair, and sit down again. The subject wears his regular footwear and uses his customary walking aid (none, cane, or walker). Three tests will be performed with each subject in a 1-min interval, with the best performance being chosen as the final measure [16].

According to the test, an execution time of less than 10 s suggests totally free and independent individuals. Individuals who perform the test between 10 and 19 s are considered independent, and those who perform the test in 20 to 29 s are in the so-called “grey zone,” that is, demonstrating limited functional capacity and difficulties in tasks of daily living. Those who perform the test in 30 s or more tend to be totally dependent for many basic and instrumental activities of daily living [16].

The measurement process of physical activity levels requires the use of reliable and valid assessment tools. One of the most common assessment tools is questionnaires, with the advantage of low cost and easy administration. To evaluate the levels of physical activity among the elderly subjects involved in this study, a well-known efficacy instrument called the Physical Activity Questionnaire for Older Adults (CHAMPS) will be used. The CHAMPS is one of the first physical activity questionnaires for older adults designed specifically for use in evaluating interventions that primarily aim to increase levels of physical activity in older adults. According to the authors, the CHAMPS measure may be useful for evaluating the effectiveness of programs aimed at increasing levels of physical activity in older adults [17].

The CHAMPS assesses the level of physical activity in older adults (> 60 years and/or) taking into consideration caloric expenditure and frequency of the activities performed. This instrument is composed of 41 questions in which the answer is “yes” or “no.” After responding “yes,” the answer specifies the frequency of days and hours per week that the activity was performed within the last 4 weeks. The types of activities assessed by CHAMPS are activities of daily living at home, exercise, and recreational activities [17, 18]. In this study, the CHAMPS will be applied at the baseline moment of data collection and at the end of the execution of the home exercise program, according to the proposed protocol.

Quality of life will be evaluated using the World Health Organization Quality of Life Group for Older Adults (WHOQOL-OLD) questionnaire, which contains six facets of four items each, evaluated by a Likert scale (1 to 5 points): Facet I **–** “Sensory Function”; Facet II **–** “Autonomy”; Facet III **–** “Past, Present, and Future Activities”; Facet IV **–** “Social Participation”; Facet V **–** “Death and Dying”; Facet VI **–** “Intimacy.” Each of the facets has four items; thus, for all facets the score of the possible values ranges from 4 to 20, and the scores of these six facets or the values of the 24 items can be combined to produce a “global” score of the QoL [19].

## Intervention

### Physical exercise program at home

The home exercise program will be based on the recommendations of the American College of Sports Medicine for exercise and physical activity of elderly people [3]. The program consists of aerobic exercises, muscle strengthening exercises, balance training, motor coordination, and flexibility, always prioritizing exercises involving large muscle groups. The protocol will last for 12 consecutive weeks, with a minimum frequency of three sessions per week and a planned execution time of 40 min. During each session, two to three sets will be performed with 5 to 15 repetitions for each exercise at a target effort rate of 13–15 (“a little difficult” to “difficult”) on Borg’s perceived exertion scale of 6 to 20 points [20].

The participant will perform the exercise program individually in their own home, without direct supervision during its execution, but with on-site guidance through home visits every 15 days by one of the members of the team. Participants will be instructed to increase the intensity of the exercises, using the Borg scale to evaluate intensity, and in a manner proportional to their execution capacity, evaluated by the research assistants at each of the visits. The exercises will be carried out using the weight of the body itself and with the aid of some low-cost equipment (recyclable plastic bottles to demarcate the signage of the course, sticks and weights of 1 and 2 kg for performing the resistance exercises). The following are the exercises to be performed:

- Warm-up exercises – Active-free exercises of the upper and lower limbs, including extension, flexion, and rotation of the shoulders associated with breathing exercises
- Aerobic exercises – Displacement of a stick with both hands, from the knees to above the head and returning to the knees, and walking exercises with alternating thigh flexion and placing the hand on the opposite knee
- Resistance exercises – For the upper limbs: starting from the position with the elbow extended and the hand resting on the opposite thigh, movement of the whole member diagonally upwards and then returning the hand to the thigh. For the lower limbs: squatting exercise, starting from the sitting position on a chair and with arms crossed in front of the body, lifting to the orthostatic position and then returning to the sitting position
- Balance and coordination exercises – Walking on a straight line and walking while diverting from lined obstacles with progressively smaller distances. When possible, the exercise will evolve and the walk will be performed by touching the heel of one foot to the toes of the other foot (foot with foot)
  Note: to ensure safety, these exercises will be performed close to fixed furniture in the house, making it possible to lean when necessary
- Stretching exercises – From the sitting position and with knees in extension, trying to reach the tip of the feet; from the sitting position on a chair and with the feet on the ground, performing rotation of the trunk to one side and elevation of the upper limb, on the same side, above the head, stretching as high as possible

During the period of the proposed program (12 consecutive weeks), regular home visits will be made to the participants of the two groups in order to clarify doubts, guide healthy living habits, and encourage adherence to the program. The IG will receive, in addition to these guidelines, specific monitoring in relation to the practice of the exercises and assistance with possible adverse events. After the end of the proposed 12-week period, the researchers will reassess the participants in both groups and encourage them to continue with the home program. Those in the CG will be provided follow-up for the regular practice of home exercises for the same period performed by the IG participants.

### Statistical analysis

The principle of intention-to-treat analysis will be respected. For missing data, we will perform a sensitivity analysis, through simple imputation, using the mean of the variables. Student’s *t* test for numerical variables and Pearson’s chi-square test for categorical variables will be applied to verify whether the randomization process generated two groups of subjects (CG and IC) with homogeneous clinical and demographic characteristics before intervention, thus avoiding a possible selection bias. To test the normality of the data, an analysis of the study histogram, mean and median, standard deviation, skewness, and kurtosis will be performed and for its confirmation, we will use the Shapiro-Wilk normality test. With a normal distribution of variables, parametric statistics will be used, and intragroup comparisons will be made using the Student’s *t* test for paired samples; the intergroup comparisons will be performed using the Student’s *t* test for independent samples. If there is no normal distribution of the variables, the corresponding non-parametric tests will be used. For the analysis of the groups by age, as will be done with more than two groups, we will use the one-way analysis of variance (ANOVA) for parametric distribution or Kruskal-Wallis for non-parametric analysis. The significance level established for all analyses will be *p* < 0.05 and all statistical procedures will be analyzed and processed using the Statistical Package of the Social Sciences SPSS 21.0 software (IBM® SPSS version 21, IBM, Armonk, NY, USA).

### Sample size

The sample size was calculated using the WINPEPI (PEPI-for-Windows) [21] software, based on the main hypothesis of the study. According to a relevant study published by Lacroix et al. [8], where a group of health older adult subjects performed unsupervised exercises obtaining a reduction of 0.37 s in TUG execution time when compared to pre and post intervention with duration of 12 weeks. Considering the standard deviation of 0.84 for the IG and 1.02 for the CG observed in the study, with a statistical power of 80% and an alpha error of 5%, 101 participants in each group would be necessary. The sample will be increased by 10% to compensate for possible dropouts, leading to 111 subjects in each group (overall sample = 222 participants).

### Outcome measures

#### Primary outcome

The expected primary outcome is the change in the functional mobility of the elderly involved in the study, verified through the mean time in minutes for the TUG test, compared at the baseline and after the 3 months’ post-intervention period.

#### Secondary outcomes

The expected secondary outcomes for the subjects involved in this study will be the change in QoL verified by the WHOQOL-OLD questionnaire and the improvement in the level of physical activity measured using the CHAMPS, compared at the beginning and after the post-intervention period of 3 months. Feasibility of the protocol will be determined by the number of eligible elderly people recruited and retention and adherence rates to the home-based exercise program. The retention rate will be defined as the percentage of subjects who completed the protocol. Adherence will be defined as the percentage of exercise sessions attended by those who were randomized to the IG. Adherence to the home-based exercise program will be recorded using attendance records (recorded by the study physicians and physiotherapists) and participant exercise diaries (recorded by the participant). The proportion of subjects distributed between the CG and the IG will be 1:1, according to the randomization process.

### Data monitoring and quality control

Systematic training of five assistants will be carried out exclusively for evaluations, and training of ten assistants will be performed for home monitoring of elderly people, five of whom will visit the IG and five of whom will visit the CG. The distribution of the number of participants to be evaluated and the number of domiciles to be visited will be done in an equivalent way among the research assistants.

### Achieving retention

Once the subject is officially registered in this trial, both in the CG and in the IG, the researchers involved in the study will make every effort to avoid possible sample losses. A number of strategies will be adopted such as weekly telephone contact; home visits every 15 days and an emergency telephone number to report any changes in the physical and/or psychological condition. In case a subject abandons the trial, this subject must be replaced by another one, remembering that in the sample calculation a sample loss of 10% in each group was considered. Adherence to the exercise protocol will be verified through the weekly records that will be filled by the participants themselves, with the help of their relatives, and will be certified by the assistants during the home visits.

### Safety

Although clinical evaluations and tests are routine in academic life, possible adverse events, highlighting falls, hospitalizations, and deaths, will be monitored and recorded by the researchers directly involved in the study and reported to the research coordinator of the study and to the Research Ethics Committee responsible for the ethical approval of the study protocol.

### Dissemination policy and data sharing

The results of this study, whether positive, negative or inconclusive, will be submitted to the peer-reviewed international journal for possible publication. Additional data are available from the corresponding author on reasonable request.

### Quality control, data management, and governance

All investigators who will participate in the study are qualified physicians and physiotherapists, including a chief physician supervisor, visiting staff, and residents of the Bahia School of Medicine and Public Health, Salvador (BA), Brazil and Bahia State University – UNEB, Department of Education (DEDC-VII), Senhor do Bonfim (BA), Brazil. In order to avoid possible biases in the collection and analysis of the data, searching for robust and high-quality results, the main investigator (GSB) and the statistician will do the manual conference and release of all the data referring to the subjects involved in this study, inserted in a single database. The data will be coded and the identity of the subjects will be preserved. The governance of the trial will be carried out by the Study Management Committee, composed of the main investigator (GSB), statistician, and physician supervisor (AAC), who will control data quality through weekly meetings and rigorous training to ensure that all researchers involved in study are following the proposed protocol. During all phases of the trial, the Study Management Committee will supervise the study ensuring that [1] the subjects recruited are within the scope of the protocol [2]; all participants meet the inclusion criteria; and [3] that all participants perform the activities proposed by the clinical trial. A performance audit will be conducted every 2 weeks of activities with the entire team of researchers.

## Discussion

This is a study designed to verify whether a semi-supervised, home-based exercise program alters the functional mobility and QoL of sedentary community elderly persons when compared to a control population. This hypothesis is based on research that has already demonstrated the efficacy of physical exercise programs in terms of functional mobility [7, 22, 23] and improving the QoL of elderly people [8, 9]. However, most of these studies adopted professional supervision during the execution of exercise programs, in addition to being performed in training and/or rehabilitation centers [24–26], which is characterized as a limiting factor for the participation of elderly people, as they may have difficulties with mobility and transfer [27]. The benefits of such programs depend on the degree of adhesion to exercise, which is influenced by the intensity of pleasure and satisfaction provided [27, 28].

It has already been demonstrated in the literature that physical exercise performed at home, in addition to being the preference of elderly people, can provide important health benefits [7, 25, 29], leading to improved adherence and continuity of the exercises after the end of the proposed program [30].

One of the main characteristics of the present study is the focus on the practice of semi-supervised exercises. A strength of this study is that, after the random allocation of the participants, both groups will receive periodic home visits with the same frequency, aiming to provide the same orientation and stimuli in relation to healthy lifestyle habits. This allow the groups to acquire similar behaviors, differing only in terms of the practice of physical exercise, considering that previous studies suggest that frequent contact with the participants by telephone calls, Internet, or personal visit increases adherence of elderly people to home exercise programs [8, 29, 30].

The lack of scientific evidence demonstrating the benefits of semi-supervised home exercise on functional mobility and QoL in elderly people represents an obstacle to the development of guidelines for clinical practice and for policy-makers. The World Health Organization highlighted the importance of musculoskeletal health programs for elderly people [31], and the exercise program described in this protocol was designed to be viable, easy to implement, and inexpensive and could be performed in elderly individuals’ homes after receiving only guidelines and follow-up via periodic visits. Based on these facts, we hope this study will demonstrate that a well-structured, home-based exercise program can be effective in improving functional mobility and QoL of sedentary elderly individuals, even without constant supervision during exercise. The results of this study should be interpreted considering some limitations. The inability to blind participants to the intervention may be mitigated by the fact that different assistants will accompany each group (CG and IG), minimizing the excitement bias applied by the IG assistants during home visits. Monitoring of the frequency of exercise completion will be self-referenced; in order to increase the reliability of this information, in addition to monitoring of the frequency register during home visits, family members will be recruited to assist in these annotations.

### Trial status

The study is currently recruiting participants. This phase is expected to end in July 2019.

## Additional files

Additional file 1: Standard Protocol Items: Recommendations for Interventional Trias (SPIRIT) Checklist. (PDF 129 kb)

## Abbreviations

BMI: Body Mass Index
CG: Control group
CHAMPS: Physical Activity Questionnaire for Older Adults
IG: Intervention group
MSME: Mental State Mini Exam
QoL: Quality of life
TUG: Timed Up & Go
WHOQOL-OLD: World Health Organization Quality of Life for Older Adults
